# The efficiency and productivity-changing trend of PHCIs since the 2009 health reform in China based on a three-stage DEA and Malmquist Productivity Index

**DOI:** 10.7189/jogh.15.04045

**Published:** 2025-02-21

**Authors:** Ling Liu, Jia Peng, Sumit Kane, Chenkai Wu, Yumei Liu, Jiayan Huang

**Affiliations:** 1 NHC Key Laboratory of Health Technology Assessment, School of Public Health, Fudan University, Shanghai, China; 2 Nossal Institute for Global Health, Melbourne School of Population and Global Health, University of Melbourne, Australia; 3 Global Health Research Center, Duke Kunshan University, Kunshan, China; 4 International School of Public Health and One Health, Hainan Medical University, Haikou, Hainan Province, China; 5 Harvard T.H. Chan School of Public Health, Harvard University, USA

## Abstract

**Background:**

In China, most primary health care institutes (PHCIs) support ground-level medical services which are essential to residents’ health levels. The Chinese government implemented a health reform in 2009 to strengthen PHCIs through increased fiscal inputs. However, how efficiently these inputs were converted into PHCIs’ services remains unclear. We aimed to examine the efficiency of PHCIs’ medical services and investigate if any changes occurred following the implementation of the health reform.

**Methods:**

We aggregated data from PHCIs from Hainan’s 18 districts (2011–21), treating those from the same district as one decision-making unit (DMU). We used three-stage data envelopment analysis (DEA) to assess the efficiencies of these PHCIs, adjusting the approach for environmental factors, managerial ineffectiveness, and statistical errors potentially arising from the background variability of measured data that deviates from the input and output values, allowing all DMUs to be compared in a homogeneous environment. We used the adjusted efficiency scores to evaluate the efficiency of PHCIs in Hainan each year and the Malmquist Productivity Index (MPI) to explore the productivity change of PHCIs over time.

**Results:**

After adjusting for environmental factors between 2011–21, technical efficiency (TE) decreased from 0.825 to 0.745, pure technical efficiency (PTE) increased from 0.936 to 0.954, and scale efficiency (SE) decreased from 0.883 to 0.783. Seven districts had full PTE (1.0) and two districts had full TE (1.0) after adjustment. The mean MPI from 2011 to 2021 was 0.9430, indicating a 5.7% decrease in PHCIs’ efficiency. After excluding the low productivity index possibly influenced by COVID-19 (2019 to 2021), PHCIs’ efficiency decreased by 0.49%, with a mean MPI of 0.9951.

**Conclusions:**

The efficiency of PHCIs in Hainan has declined slightly since the health reform. Low level of scale efficiency posed a significant impact on the overall efficiency of the medical services in PHCIs. Among potential inefficient technological performances, future policy formulation might focus more on the imbalanced allocation of resources in less-developed regions and PHCIs’ lack of attractiveness to local patients.

The concept of primary health care (PHC) is widely regarded as a key strategy for ensuring health equity [1]. From the Alma-Ata Declaration (1978) [1] to the Declaration of Astana (2018), the types of services that primary health care institutions (PHCIs) are expected to deliver have expanded to include those addressing general population health issues, including disease prevention, preliminary diagnosis and referral, health promotion, and rehabilitation [3–7]. When PHCIs provide quality care, they can play a crucial role in achieving universal health coverage (UHC) and the Sustainable Development Goals (SDGs) [8].

China, like many other countries, has gradually been prioritising its primary health care (PHC) services since 2009. Through its 2009 health care reform initiative, the Chinese Government increased funding for PHCIs with the aim of improving the accessibility of essential public health services and increasing the utilisation of PHC. By 2019, it had invested over CNY 215 billion (approximately USD 32.5 billion; CNY 1 = USD 6.62 as of 2018 [9]) in PHCIs. After the reform, PHCIs were expected to support higher-level hospitals by addressing the population’s basic health needs and retaining them at the primary level. However, it still remains unclear how efficiently the fiscal and human investments have been converted into PHC services. Therefore, a timely assessment of the efficiency of PHCIs is necessary to guide future resource allocation and policy improvements. Since the progress of reforms varies greatly among provinces due to significant economic and health disparities, a better understanding of efficiency is crucial for guiding further reform policies and resource allocation.

In China, the responsibilities of PHCIs vary across regions. In economically developed areas with sufficient resources, like Beijing and Shanghai, PHCIs mainly focus on basic public health services such as disease screening and outpatient care, while in other, resource-limited regions, they are additionally responsible for medical and curative services [6,10,11]. However, they are an essential pillar of the health system in both contexts [12]. While the efficiency and quality of services delivered by PHCIs directly affect population health, they also affect the work done by the upper-level health providers to whom PHCIs refer patients. Therefore, strengthening PHCIs can help achieve UHC and desirable population health outcomes via different pathways.

Hainan is the smallest, southernmost, and one of the less economically developed provinces of China. Its peripheral, geographically flatter regions are more economically developed, while the remote, mountainous, but central ones are less so. The district’s PHCIs consist of community/township health service stations, health service centres, village clinics, and village hospitals, all of which play a key role in providing medical services by providing basic diagnosis, treatment, and inpatient care for residents for one hundred seventy categories of diseases. Additionally, unlike other provinces with a ‘province-city-county’ administrative system, counties in Hainan come directly under the province, *i.e.* do not depend on the city level. This simplified political hierarchy allows for straightforward administrative interventions from the province directly to counties and thus reduces the possibility that the effects of multiple policies could co-occur, enabling a more accurate and intuitive investigation of outcomes of the health care reform. Yet while existing studies have examined the efficiency of allocation of resources at the regional level or across multi-level cities, or have focussed on the larger, more prosperous part of the country, few have looked at regions with low health resources and low levels of economic development [13–15]. Therefore, taking Hainan as a case province, we aimed to identify potential constraints faced by PHCIs in similar, less developed regions with a view to inform policy and programme improvement initiatives. To do this, we used data envelopment analysis (DEA) models to evaluate the efficiency and changes therein in care provision in Hainan’s PHCIs from 2011–21 in order to assess the impact of the health care reform. Based on the evaluation, we attempt to discuss potential determinants associated with these inefficiencies and, while considering the generalisability of our findings, propose feasible options for improvements to other contexts that face similar challenges.

## METHODS

### Data source

Official routine service/administrative data collected by all PHCIs in Hainan Province from 2011–21 were shared by the Hainan Provincial Health Department through its governmental annual PHCI reports, while official demographic data on Hainan were shared by the Hainan Provincial Bureau of Statistics through the Hainan Statistical Yearbook for 2011–21. We considered the basic reliability and integrity of these data sets to be high, as come from official sources. For our analysis, we summed the inputs, outputs, and environmental variables from PHCIs in the same district, treating each district as one decision-making unit (DMU). Based on data availability, we included all 18 districts (DMUs) in this study: Haikou, Sanya, Wuzhishan, Wenchang, Qionghai, Wanning, Dingan, Tunchang, Chengmai, Lingao, Danzhou, Dongfang, Ledong, Qiongzhong, Baoting, Lingshui, Baisha, and Changjiang. As the collected data were deidentified and anonymised prior to our use, and as we collected no new data or biological samples, we did not require ethical approval.

### Input and output indicators

We used five inputs and three outputs to evaluate the efficiencies of all PHCIs in the 18 districts. There are three types of inputs in PHCIs: human resources, material resources, and financial investment. PHCIs’ staffing arrangements were taken to represent the human resource inputs [14,16,17]. Annual bed-days, building area, and equipment above CNY 10 000 served as a proxy for material resources. Financial allocation for medical services was taken to represent the financial inputs. Guided by the national requirements for service delivery in PHCIs, the number of visits, admissions, and discharged patients were taken as output variables [13]. Prior studies suggested that the number of DMUs should be more than twice the number of input and output indicators together [18]. Here we had 18 DMUs >2 × (5 inputs +3 outputs) = 16, which means we met these criteria.

### Environmental factors

Based on all available demographics in the Hainan Statistical Yearbook (2011–21) [20–24] and existing research [20–24] , we adjusted for the following six environmental factors due to their potential influence on efficiency scores: population density, the proportion of individuals aged >65 years, urbanisation, the proportion of ethnic minorities, the log of regional gross domestic product (GDP), and per capita GDP (Table S1 in the **Online Supplementary Document**).

Population density was shown to be related to government management and supervision costs, regions’ social resources, and environmental development [20], so we assumed they could affect resource allocation and operation of PHCIs in different regions. Additionally, it was also found to be associated with different disease burdens, potentially affecting PHCI utilisation in different regions [21]. In prior research, the proportion of individuals aged >65 represented the impact of ageing on the operating efficiency of the PHC [22]. China is one of the countries with the fastest-growing ageing population in the world; 15.4% of its population was aged >65 years in 2023, and this percentage is projected to reach 26.1% by 2050 [23]. Ageing entails higher risks of chronic non-communicable diseases, increasing demands and the utilisation of PHC [24]. Levels of urbanisation had previously been included as a proxy for social capital, local employment, financial subsidies, and individual contributions [25]. This clustering effect could attract more people from the rural to the urban regions, resulting in a decline in PHCI utilisation in the former and an increase in the latter. We adjusted for the proportion of ethnic minorities due to the potential impact of different cultural traditions on the utilisation of PHCs. For example, some ethnic minorities seek help from religious rituals and traditional herbal medicines rather than from medical institutions.

Guided by World Health Organization (WHO), we used the total GDP to characterise regional macroeconomic development level regardless of allocation, and per capita GDP to reflect average living standards and residents’ economic well-being [25]. Relying on local financial appropriation, the operation of PHCI is impartible from the support of local government. The macroeconomic development level, the region’s overall health, and economic well-being are all associated with the government’s allocation of health expenditures.

We conducted the descriptive analysis of input indicators, output indicators, and environmental factors in Python, version 3.19. 13 (Python Software Foundation, Wilmington, Delaware, USA).

### Three-stage DEA

Data envelopment analysis (DEA) models, which can measure the efficiency of units with multiple inputs and outputs, have been widely used to evaluate the efficiency of health services. They can broadly be categorised as input-oriented and output-oriented. Previous studies employed input-oriented DEA models to analyse the efficiency of health centres and investigate determinants affecting hospitals’ activities [14,26,27]. Output-oriented DEA models have been used to measure the efficiency of maternal and child health resource allocation [28]. In addition, they have been applied in three different ways – as traditional, two-stage, and three-stage DEA models. For our analysis, we employed the three-stage DEA model, which puts all the units in a homogeneous environment for comparison and yields bias-adjusted efficiency, meaning that it adjusts for statistical errors, bias, and potential impacts of environmental factors that the other two models fail to consider.

For our research, the three-stage DEA model measured the productive efficiency of DMUs and included conventional input-oriented DEA efficiency analysis; stochastic frontier analysis (SFA) to decompose environmental influences, managerial inefficiencies, and statistical noises, as well as input and output measurement errors; and efficiency re-evaluation with adjusted inputs.

To ensure the robustness of DEA, we further performed sensitivity analyses by altering the input and output indicators. We discarded one input indicator each time and employed DEA with the remaining four inputs and the original three outputs. Similarly, we discarded one output indicator each time and employed DEA with the remaining two outputs and the original five inputs. In this way, we set up eight DEA models in total. We otherwise used the Malmquist Productivity Index (MPI) to analyse efficiency comparisons between different years as DEA models only yield point efficiency scores for each year.

We used *R*, version 4.0.5 (R Core Team, Vienna, Austria) and its ‘benchmarking’ (stage 1 and 3), ‘frontier’ (stage 2), and ‘deaR’ (Malmquist Productivity Index (MPI)) for our analyses.

#### Stage 1: input-oriented DEA

The orientation of the model should be clarified before performing DEA. Input-oriented models focus on the extent to which each input should be reduced to achieve efficiency with constant output, while output-oriented ones focus on the extent to which each output should increase to be effective with constant input. As inputs for PHCIs are more controllable compared to outputs, we conducted a conventional input-oriented DEA using input and output data. Existing implementations of DEA measure DMUs’ efficiency as technical efficiency (TE), which can be decomposed into pure technical efficiency (PTE) and scale efficiency (SE), per the formula *TE* = *PTE* × SE. Here, TE measures the overall performance, PTE reflects the allocation and management efficiency, and SE measures the gaps between the current and optimal production scale. All efficiencies range between 0 and 1. A DMU becomes more efficient as its efficiency approaches 1.

#### Stage 2: SFA

In the second stage, we used SFA to adjust slacks from stage 1 for influences of environmental factors, random disturbances, and managerial inefficiencies. We used input slack variables as explained variables and six environmental factors (population density, proportion of elderly aged >65 years, urbanisation rate, proportion of ethnic minorities, GDP, and per capita GDP) as explanatory variables. We estimated the influences of the environmental factors using regression analysis under the maximum likelihood estimation (MLE). The SFA is expressed as *S_ik_* = *f_i_*(*Z_k_*; *β_i_*) + *v_ik_* + *u_ik_*, where *s_in_* is the slack variable of k^th^ (k = 1, 2…m) DMU’s i^th^ (I = 1, 2…n) input; *f_i_*(*z_k_*;*β_i_*) is the effect of environmental factors on the slack variable; *z_k_* is the environmental factor for the k^th^ DMU; *β_i_* is the coefficient of environmental factors for the i^th^ input; *v_ik_* + *u_ik_* is the error term; *v_ik_*  is the random disturbances and is normally distributed with *v_ik_* ~ *N* (0,*σ^2^_vi_*); *μ_ik_* is the managerial inefficiencies.

Assume *μ_ik_* follows a truncated normal distribution as *u_ik_* ~ *N* (0,*σ^2^_vi_*), and *μ_ik_* is independent of *v_ik_*. Let γ  = *σ^2^_vi_*/(*σ^2^_ui_* + *σ^2^_vi_*). If random disturbances dominate more of the error term *v_ik_* + *u_ik_*, γ approaches 1; if managerial inefficiencies take the dominant role, γ approaches 0.

As input slack variables can be viewed as input redundancies, a positive coefficient established by environmental factors suggests increased input redundancy and reduced PHCI efficiency. Negative coefficients suggest decreased redundancy and increased efficiency. Here we used a one-sided generalised likelihood ratio test for validation. If each regression was significant at a 1% significance level, we considered the application of the SFA model to be reasonable.

#### Stage 3: DEA with adjusted efficiency scores

At this stage, we adjusted all original input variables used in stage 1 with the above SFA regression to ensure that DMUs are under the same external environment. The adjustment equation was *x^A^_ik_* = *x^A^_ik_* = [max(*f_i_*(*z_k_*;*β_i_*)) − *f_i_*(*z_k_*;*β_i_*)]+ [max(*v_ik_*) − (*v_ik_*)], where *x^A^_ik_* is the adjusted i^th^ input of k^th^ DMU; *x_ik_* is the original i^th^ input of k^th^ DMU used in stage 1; [max(*f_i_*(*z_k_*;*β_i_*)) − *f_i_*(*z_k_*;*β_i_*)] eliminates influences of environmental factors and adjusts all DMUs to the same external environment; and [max(*v_ik_*) − (*v_ik_*)] adjusts randomdisturbances for all DMUs. We estimated the influences of the environmental factors were estimated using regression analysis under the MLE. Then, after all the input variables in stage 1 are adjusted for, *x^A^_ik_* are used to recalculate TE, PTE, and SE, repeating the conventional input-oriented DEA analysis in stage 1. These adjusted efficiency scores allow the evaluation of productive efficiencies after adjusting for potential environmental influences.

#### MPI

Considering DEA can only measure the efficiency in a certain period, the MPI approach is used to uncover longitudinal changes in efficiencies. First introduced in 1953, non-parametric MPI allows the comparison of efficiency changes over time [29]. Based on different assumptions of returns to scale, we followed decompositions of input-oriented MPI to understand various sources of efficiency changes. The formula for MPI follows:



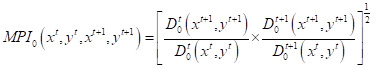



where *x* and *y* are the inputs and outputs in *t* or *t +1* period, and where 1) and 2) below use distance function to respectively measure technology at period *t* and *t +1*.



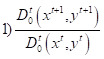





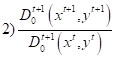



According to Fare’s analysis in 1994, MPI can be further decomposed into technological change (TEch) and efficiency change (EFFch). Then EFFch can be further decomposed into pure efficiency change (PEch) and scale efficiency change (SEch). Thus, a full decomposition of MPI is suggested as *MPI* = *TEch × EFFch = TEch × PEch × SEch.* Here, *TEch* reflects the changing index of efficiency frontier and *EFFch* reflects the changing index of efficiency performances. Values of *MPI* can be smaller, equal to, or greater than 1, respectively indicating productivity growth, stagnation, and decline.

## RESULTS

### Overview of PHCIs and environmental factors

#### PHCIs’ inputs and outputs characteristics

After treating all PHCIs in the same district as one single DMU, we included 18 districts (DMUs) in Hainan from 2011–21. We observed improvements from the input side over these ten years. The mean number of staff per DMU increased from 586 people to 986 people. The mean annual number of bed days available held by PHCIs per DMU increased from 113 000 to 176 000. The mean building area per DMU increased from about 36 500 m^2^ to over 76 000 m^2^. The mean amount of equipment with a value above CNY 10 000 (USD 1366) per DMU increased from 88 to 339. The mean financial allocation for medical services per DMU increased from CNY 12.95 million (USD 2 million) to nearly CNY 59.29 million (USD 9.19 million). We observed no such increasing trend on the output side. The mean number of visits per DMU first increased from 607 000 in 2011 to over 829 000 in 2019 and then decreased to about 688 000 in 2021. The number of admissions and discharged patients per DMU fluctuated around 5500 and 5900 from 2011 to 2017 and decreased to around 3000 in 2021 (Table S2 in the **Online Supplementary Document**).

#### Environmental factors characteristics

The average population per DMU in Hainan increased from 487 400 in 2011 to 566 800 in 2021. The average population density per DMU increased from 256.55 to 296.24 per km^2^. The average proportion of elderly aged >65 years per DMU increased from 8.08% to 10.78%. The urbanisation rate per DMU increased from 50.5% to 60.97%. The proportion of ethnic minorities fluctuated around 16.67% in the study period. The average GDP per DMU almost tripled in these years, *i.e.* increased from CNY 13.97 billion (USD 2.16 billion) to CNY 35.95 billion (USD 5.57 billion). The average per capita GDP per DMU in Hainan increased from CNY 28 790 (USD 4460) to CNY 63 670 (USD 9870). (Table S4 in the **Online Supplementary Document**)

### Adjusted PHCIs efficiencies

The changes in efficiency scores and disparities among different DMUs in the sensitivity analysis were consistent with the main results, suggesting that the DEA was robust (Table S5–8 in the **Online Supplementary Document**). After eliminating the environmental influences and managerial inefficiencies for all 18 districts in Hainan from 2011 to 2021, the adjusted average TE, PTE, and SE were 0.745, 0.954, and 0.783, respectively (**Table 1**, **Table 2**).

**Table 1:** Mean efficiency scores and ranges of PHCIs in Hainan 2011–21 by changing years

	Conventional (stage 1)			SFA adjusted (stage 3)		
**Year**	**TE (range)**	**PTE (range)**	**SE (range)**	**TE (range)**	**PTE (range)**	**SE (range)**
2011	0.8200 (0.4454–1)	0.9275 (0.4975–1)	0.8893 (0.4454–1)	0.7420 (0.2496–1)	0.9556 (0.5716–1)	0.7811 (0.2496–1)
2012	0.7911 (0.3701–1)	0.8933 (0.4450–1)	0.8911 (0.4164–1)	0.7111 (0.2527–1)	0.9224 (0.5472–1)	0.7775 (0.2527–1)
2013	0.8012 (0.3518–1)	0.9006 (0.4686–1)	0.8909 (0.4053–1)	0.6952 (0.2423–1)	0.9327 (0.5420–1)	0.7545 (0.2423–1)
2014	0.7805 (0.3820–1)	0.8833 (0.4718–1)	0.8849 (0.4249–1)	0.6776 (0.2644–1)	0.9265 (0.5292–1)	0.7382 (0.2258–1)
2015	0.8265 (0.4513–1)	0.9239 (0.5872–1)	0.8955 (0.4808–1)	0.7183 (0.3108–1)	0.9403 (0.6188–1)	0.7666 (0.3347–1)
2016	0.8565 (0.4187–1)	0.9541 (0.6641–1)	0.8959 (0.4740–1)	0.7821 (0.3380–1)	0.9615 (0.7224–1)	0.8118 (0.3692–1)
2017	0.8516 (0.4326–1)	0.9573 (0.7981–1)	0.8885 (0.4836–1)	0.7960 (0.3654–1)	0.9728 (0.7931–1)	0.8201 (0.3654–1)
2018	0.8744 (0.4653–1)	0.9659 (0.7758–1)	0.9055 (0.4653–1)	0.7869 (0.2474–1)	0.9887 (0.8351–1)	0.7975 (0.2474–1)
2019	0.8700 (0.4121–1)	0.9854 (0.8892–1)	0.8834 (0.4121–1)	0.8092 (0.2334–1)	0.9902 (0.8920–1)	0.8181 (0.2334–1)
2020	0.8328 (0.3496–1)	0.9597 (0.6022–1)	0.8718 (0.3496–1)	0.7524 (0.2218–1)	0.9553 (0.7349–1)	0.7836 (0.2218–1)
2021	0.7707 (0.3176–1)	0.9447 (0.6368–1)	0.8170 (0.3176–1)	0.7210 (0.1799–1)	0.9462 (0.6811–1)	0.7604 (0.1799–1)
Mean	0.8250 (0.4114–1)	0.9360 (0.6816–1)	0.8831 (0.4443–1)	0.7447 (0.2719–1)	0.9538 (0.7151–1)	0.7827 (0.2719–1)

PHCIs – primary health care institutes, PTE – pure technical efficiency, SE – scale efficiency, TE – technical efficiency

**Table 2:** Results of stage 2: SFA coefficients for analysis adjusted environmental factors

Variables	Staff (people)	*P*-value	Annual bed-days as beds × 360 days	*P*-value	Building area in m^2^	*P*-value	Number of equipment above CNY 10 000	*P*-value	Medical appropriation income (CNY 1000)	*P*-value
Intercept	−92 1788.84 (6.85)	<0.001	−276 227.76 (15.21)	<0.001	446.16 (53.6)	<0.001	−3135.07 (1.01)	<0.001	138 506.46 (30.42)	<0.001
Population density in persons/km^2^	126.37 (60.49)	<0.05	−30.96 (15.99)		−0.23 (0.0)	<0.001	1.35 (0.21)	<0.001	−11.38 (27.36)	
Proportion of aged ≥65 (%)	2656.7 (49.85)	<0.001	576.51 (184.24)	<0.01	−1.06 (0.25)	<0.001	−56.39 (2.28)	<0.001	−1179.57 (334.25)	<0.001
Proportion of urbanisation (UP/TP), (%)	−1977.2 (234.19)	<0.001	−697.2 (137.76)	<0.001	5.55 (0.17)	<0.001	−12.28 (1.98)	<0.001	626.5 (251.83)	<0.05
Proportion of ethnic minorities, (%)	593.16 (301.83)	<0.05	253.59 (48.99)		−2.47 (0.06)	<0.001	0.16 (0.59)		−299.89 (113.62)	<0.01
log(GDP (CNY 10 000))	−6350.06 (89.11)	<0.001	26 030.35 (179.22)	<0.001	49.84 (6.63)	<0.001	−138.29 (1.98)		−11 331.41 (363.48)	<0.001
log(per capita GDP (CNY 10 000))	103 387.95 (67.3)	<0.001	−4410.37 (135.78)	<0.001	−118.27 (3.84)	<0.001	582.59 (1.63)		2465.24 (278.82)	<0.001
*δ^2^*	1 402 231 852.57 (1.0)	<0.001	56 695 609.42 (1.0)		2712.36 (38.67)	<0.001	20 509.51 (1.0)	<0.001	181 154 607.78 (1.0)	<0.001
γ	0.82 (0.01)	<0.001	0.74 (0.02)	<0.001	1.0 (0.0)	<0.001	1.0 (0.0)	<0.001	0.76 (0.02)	<0.001
Log likelihood	−417.6797031		−495.8169726		−154.0210316		−198.7698331		−469.1510431	
One-sided LR test	12.379	<0.001	11.015	<0.001	13.373	<0.001	10.505	<0.001	9.725	<0.001

GDP – gross domestic product, LR – likelihood ratio, UP – urban population, TP – total population

**Figure 1:**
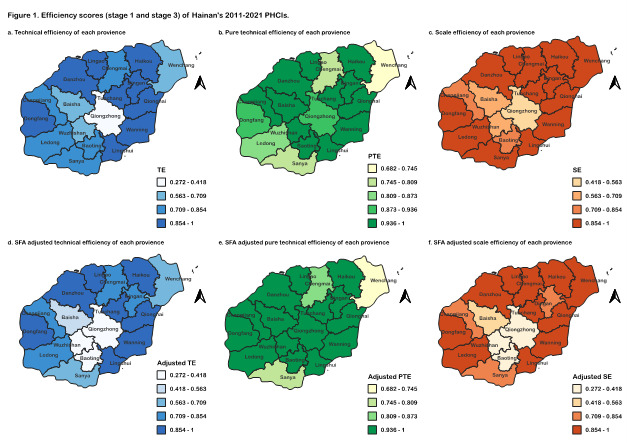
Efficiency scores (stage 1 and stage 3) of Hainan’s 2011–21 PHCIs. PHCIs – primary health care institutes, PTE – pure technical efficiency, SE – scale efficiency, SFA – stochastic frontier analysis, TE – technical efficiency.

We found great disparities among districts (**Figure 1**, Panels A–C; Table S9 in the **Online Supplementary Document**). In the 10 years, the lowest TE scores established by 18 districts always fluctuated around 0.25–0.35, with an especially low score of 0.1799 in 2021 (**Table 1**). The differences between the highest and lowest TE scores slightly decreased between 2015 to 2017 and then gradually increased, indicating persistent efficiency gaps among districts (Table S10 in the **Online Supplementary Document**). Danzhou and Lingshui maintained a full TE of 1.000 for 11 years. Wuzhishan had the lowest TE, with a score of 0.272.

For PTE, seven districts (Haikou, Wuzhishan, Qionghai, Tunchang, Danzhou, Lingshui, and Changjiang) maintained full efficiency of 1.0. The lowest PTE was established by Wenchang with a score of 0.715.

SE scores ranged from 0.272 (Wuzhishan) to 1.000 (Danzhou). Eight districts did not reach the average province TE level (Sanya, Wuzhishan, Wenchang, Chengmai, Qiongzhong, Baoting, Baisha, and Changjiang). Sanya, Wenchang, Chengmai, Lingao, and Ledong lagged on PTE, while the other districts lagged on SE. Compared to PTE, which showed relatively high scores in most regions, SE scores in most regions were farther away from 1.0, suggesting more room for improvement than PTE.

### Productivity change over the years

From 2011 to 2021, all 18 districts in Hainan established an average TC of 0.9407 and an EFFch of 1.0025, resulting in an overall MPI of 0.9430. The average SEch and PEch for the same period were 1.0032 and 0.9993, respectively, suggesting that the efficiencies remained virtually unchanged and that most MPI changes came from the TEch. Considering potential influences caused by the COVID-19 pandemic, we discarded changes during the last three years (2019–21). The resulting MPI of 0.9951 from 2011–19 still indicated a slight efficiency retrogression.

At a year-wise level, we found <1 MPI in most annual periods, except 2012–13, 2016–17, 2018–19, and 2020–21, which had MPIs of 1.0286, 1.0432, 1.0403, and 1.0367, respectively, indicating improvements in MPI of 2.86%, 4.32%, 4.03%, and 3.67%. In other years, MPI ranged from 0.5620 to 0.9902. The MPI in 2019–20 was 0.5620, suggesting a significant deterioration of 43.80%, with degradations in TEch (0.6257) of 37.43% and in EFFch (0.8982) of 10.18% (**Table 3**).

**Table 3:** MPI of Hainan’s PHCIs 2011–21 by changing years

Year	MPI (A = B × C)	TEch (B)	EFFch (C = D × E)	SEch (D)	PEch(E)
2011–12	0.9902	0.9487	1.0437	1.0567	0.9877
2012–13	1.0286	1.0326	0.9962	0.9707	1.0262
2013–14	0.9733	1.0081	0.9655	0.9562	1.0097
2014–15	0.9832	0.9050	1.0864	1.0490	1.0356
2015–16	0.9961	0.9331	1.0675	1.0497	1.0169
2016–17	1.0432	1.0660	0.9786	0.9742	1.0045
2017–18	0.9059	0.9265	0.9778	0.9688	1.0093
2018–19	1.0403	1.0547	0.9864	0.9865	0.9998
2019–20	0.5620	0.6257	0.8982	0.9560	0.9395
2020–21	1.0367	0.9980	1.0388	1.0741	0.9671
x̄	0.9430	0.9407	1.0025	1.0032	0.9993
x̄ with 2019–21 excluded)	0.9951	0.9843	1.0128	1.0015	1.0112

EFFch – efficiency change, MPI – Malmquist Productivity Index, PEch – pure efficiency change, PHCIs – primary health care institutes, SEch – scale efficiency change, TEch – technological change, x̄ – mean

**Figure 2:**
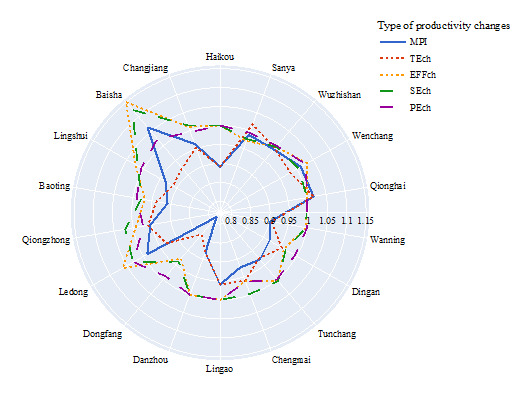
Productivity changes by district in Hainan 2011–21. EFFch – efficiency change, MPI – Malmquist Productivity Index, PEch – pure efficiency change, SEch – scale efficiency change, TEch – technological change.

At the region level, we observed improvements in MPI in three DMUs (Baisha, Qionghai, Wenchang), while the others ranged from 0.7844 to 0.9887. We further observed improvements in TEch in two DMUs (Sanya, Qionghai), while others ranged from 0.8452 to 0.9910. Lastly, we saw significant improvements in EFFch in four DMUs (Ledong, Lingshui, Baisha, Wenchang), while the others ranged from 0.9281 to 1.0000 (**Figure 2**; Table S11 in the **Online Supplementary Document**).

## DISCUSSION

Here we investigated the efficiency of PHCIs in Hainan through a three-stage DEA model. In the context of PHCIs, PTE focusses on conversion of fiscal and human resources to outputs regardless of the PHCI’s production volume and operational scales. In this context, SE measures how well a PCHI’s operation and size align with its optimal production level, while TE comprehensively considers both the pure resource conversion aspects and operational scale effects. With TE ranging widely from 0.68 to 0.81, we detected significant regional efficiency disparities. Our data show that the suboptimal technical efficiencies of PHCIs were mainly driven by their poor SE. This aligned with previous studies that show that scale optimisation was one factor hindering the total factor productivity growth in rural health sectors [15,30]. The poor SE from our analysis indicated that the medical services provided by PHCIs in Hainan did not reach the optimal scale.

### Suboptimal resource allocation in less developed regions influenced operations

We saw relatively low SE performance in PHCIs in most regions in Hainan, particularly in the poorer parts of Hainan with lower GDPs (*e.g.* Wuzhishan), where their overall efficiencies were significantly dragged by their low SE scores.

Optimal resource allocation plays a major role in achieving returns to scale [31]. Although increasing resources have been allocated to PHCIs in Hainan, significant allocation disparities exist within PHCIs among its different regions [32]. Since the responsibility for allocation of resources to PHCIs largely lies with local governments, most financial, material, and human inputs are concentrated in more economically developed regions such as Haikou and Sanya, whereas the middle part of Hainan, which has worse economic and health conditions, receives an insufficient amount of resources [33]. After adjusting for environmental factors, we specifically explored efficiency differences among Danzhou, Lingshui, and Wuzhishan. Of note, Danzhou and Lingshui are coastal counties with developed tourism industries and infrastructure investments from the government, with GDPs ranking high in Hainan [34]. Wuzhishan, meanwhile, lies in the mountainous region; its GDP has remained the worst in Hainan since 2010, while its population has been the lowest in Hainan for 10 years [34]. With the low GDP and population in Wuzhishan, allocation of health expenditures (PTE) and utilisation of PHCIs (SE) both decrease, suggesting overall TE inefficiencies. Upon this point, without sufficient support from the provincial government or better and equitable redistribution of resources, service delivery and quality of health care in these regions will enter a vicious cycle and continue to stagnate. Consequently, residents in these regions utilise local PHCIs less, which translates into lower levels of efficiency.

Simultaneously, how to productively use existing resources is critical. In this sense, PTE could be improved if fewer doctors and nurses are included in forming one treatment plan, while SE can be improved if residents with medical needs could be precisely identified and satisfied at the PHC level. However, this situation will likely continue the absence of efforts to address the inefficient use of existing inputs and inequitable financial and human resource allocation.

### The lack of continuous technological advances exacerbated the loss of competitiveness

One potential reason for Hainan’s low PHCI utilisation relates to technological insufficiencies in the province. Specifically, we observed no significant reduction in technical efficiency differences over the 10 years, although our results show a decline in technological change of over 35% from 2019 to 2020 and a continued downtrend since. Together with the health reform, China has seen rapid developments in the internet and digital transformation of health services. For instance, by using smartphones and specialised apps, patients can conveniently make appointments to see doctors, check the test results online, and so on, while doctors can track the whole health record of patients with this smart online health information system. Although these transformations have been implemented more broadly, they have largely been oriented toward hospitals in Hainan.

In some PHCIs outside of Hainan, the introduction of artificial intelligence (AI) systems for reading films or scans obtained form computerised tomography, magnetic resonance imaging, and similar devices could suggest tentative diagnoses and assist doctors in PHCIs to make treatment plans faster, increasing the PTE. This also makes up for PHCIs’ shortages in staff and professionalism. With more precise diagnosis and treatment, PHCIs win more trust from patients and chances to retain them at primary levels, increasing the SE. Besides film reading, some PHCIs utilise smart health systems which connect to higher-level hospitals. Others offer routine checks and visits by accurately tracking patients with chronic diseases who need more intensive care than one-time treatment, retaining them at the primary level, increasing the SE. However, these technological improvements are yet to be delivered at the PHCIs in Hainan, inducing gaps in its pure technological efficiencies. Rather than go to PHCIs, patients in Hainan find it easier and more convenient to visit hospitals where mobile-enabled smart services have been rolled out. Without continuous technological upgrades, PHCIs would face a loss of competitiveness and a widening of service gaps between higher-level hospitals. This gap would result in them failing to attract patients, and over time, potentially induce further declines in scale efficiencies.

Furthermore, PHCIs are challenged by and are struggling with even more such declines. According to our experience, due to a lack of a gatekeeping mechanism in China and due to people’s mistrust, PHCIs find it difficult to retain patients, as they can very easily shift from seeking local PHC services to directly consulting at higher-level hospitals. From our findings, we see that the shift of patients lowered the utilisation and operation of PHCIs and subsequently decreased the SE. Simultaneously, the much-improved transportation infrastructure has enabled patients to have more choices of health services. In 2012, 50% of beds in Shanghai’s well-known tertiary hospitals were being used by patients from other provinces [35]. Moreover, decreased medical costs for cross-provincial patients also stimulated patients’ shifting choices. In 2021, China recorded 4.459 million cross-provincial instant resettlements in a single year [36]. Convenience and affordability of cross-province visits increase the propensity of residents to seek medical care in higher-level hospitals, which further decreases patients’ utilisation of PHCIs.

Therefore, initiating technological upgrades and integration into PHCIs is crucial in managing existing input resources. In the context of Hainan, technological deficiencies could be offset through the integration of AI reading in MRI and CT, as well as sophisticated digital health systems linked to higher-level hospitals, such as the one for tracking patients which we mentioned above. Increased utilisation of technologies facilitates the reallocation of fiscal and human resources. In this way, funding and personnel can be shifted to positions that require extensive human power and are irreplaceable by advanced technologies. Furthermore, the development of health systems enhances PHCIs’ ability to retain patients at primary levels, laying a concrete foundation for future formulation of a feasible and effective gatekeeper mechanism.

### Poorly defined responsibilities of PHCIs undermined efficiency recovery after COVID-19

From 2019 to 2020, the number of visits, admissions, and discharged patients in PHCIs decreased by over 25%. We contend that this can be attributed to PHCIs’ stagnation during the COVID-19 pandemic. Given that PHCIs have a modest financial base and limited material and human resources, the central government of China regarded them as not well-placed to play a major role in the pandemic response and thus halted the operations of PHCIs during the pandemic. Therefore, designing and clarifying the function of the PHCIs could also affect their efficiency.

The COVID-19 pandemic introduced significant changes to PHCIs in Hainan; during the pandemic, the district experienced a dramatic decrease of over 45% in its Malmquist productivity. Specifically, PHCI’s responsibilities changed to focussing on finding suspected COVID cases and reporting them to higher hospitals [37]. As China prioritised the treatment and control of COVID-19, fiscal inputs, physical materials, and medical staff at PHCIs were resupplied and reassigned to collect samples for COVID-19 testing, distributing supplies, and so on, leading to a decline in PTE. Moreover, many PHCIs were shut down during the pandemic, which drastically decreased the SE. The ongoing management of many non-communicable diseases such as hypertension, diabetes, cancer, and so on. was significantly disrupted [38]. This was a common experience globally –health workers who treated patients with noncommunicable diseases were reassigned to support COVID-19 [38]. The PHCIs services have yet to be fully reactivated post-pandemic, as seen by an overall productivity index increase of only 3.67%.

### The value of investing in strengthening PHC

Global evidence suggests that investments in improving and strengthening primary care services can translate into improvements in the efficiency, effectiveness, and equity of the whole health system [39–41]. In fact, there is robust evidence to support that the highest-performing health systems in the world are those that have a strong primary care system that is well integrated with and works in harmony with the higher level of care [42]. Where primary care services are weak and where they are not well integrated and aligned with higher levels of care, the efficiency, effectiveness, and equity of the whole health system are undermined [39,43]. Prior research has provided guidance to countries on investments in interventions to improve primary health care, they argue that ‘needs to be explicitly identified, and plans should be made for how to most appropriately reorient the health system towards PHC as a key lever towards achieving UHC and the health-related SDGs’ at country level [44,45]. In terms of economic development, Hainan is at the midpoint of all provinces in China and thus faces challenges around inequitable allocation and distribution of human and financial resources that are typical to those of other provinces with similar development levels and complicated geographic compositions. Considering that the limited resources and grim economic situations are issues faced by nearly all other countries, how to efficiently utilise available allocations is crucial [46].

### Limitations

We should note several limitations of our study. First, though we have included all available relevant environmental factors to minimise their effects on efficiencies, we had to leave out some relevant environmental factors due to a lack of data and could not adjust for their influences. Second, though we have performed sensitivity analyses to suggest the robustness of our model, there is generally a lack of guidance on misspecification or parametric statistical methods to evaluate DEA model qualities [48]. Additionally, the formation of the efficiency frontiers in DEA models is sensitive to the inputs, outputs, and potential outliers of data [49]. Third, limited by budgets, we were not able to conduct qualitative research that would help us understand the root causes of inefficiencies in PHCIs at a deeper level.

## CONCLUSIONS

The efficiency of PHCIs in Hainan can still be improved. Further explorations of this challenge can include qualitative investigations, additional environmental factors, and correlations between specific allocation inputs and efficiency changes to guide the sustainability of PHC investments. Constrained by limited and imbalanced allocation of resources, progress in improving PHCIs’ efficiency has been slow. The poor coverage of mobile and other technologies and the lack of gatekeeper mechanisms are further impeding scale optimisation. Prioritising technological advancements in this context is, therefore, essential. Through the integration of advanced technical assistance, future allocation of financial and human resources can be optimised and feasible and effective gatekeeper mechanism established to boost the functioning and efficiency of primary health care services in rural remote parts of China and other districts and countries facing similar challenges like Hainan.

## Additional material


Online Supplementary Document
