# Supplementary material for: The efficiency and productivity-changing trend of PHCIs since the 2009 health reform in China based on a three-stage DEA and Malmquist Productivity Index
Source: J Glob Health. 2025 Feb 21;15:04045. doi: 10.7189/jogh.15.04045 (PMC11893142; doi:10.7189/jogh.15.04045)
Supplement: Online Supplementary Document [file jogh-15-04045-s001.pdf]

Supplement to: Liu L, Peng J, Kane S, Wu C, Liu Y, Huang J. The efficiency and productivity-changing trend of PHCIs since 2009 Health Reform in China: based on a three-stage DEA and Malmquist Productivity Index. J Glob Health 2024;15:04045.

ONLINE SUPPLEMENTARY DOCUMENT

The Efficiency and Productivity-changing Trend of PHCIs since 2009 Health Reform in China: Based on A Three-stage DEA and Malmquist Productivity Index.

Ling Liu, MFin[1,5]*;

Jia Peng[1]*,

Sumit Kane Ph.D.[2],

Chenkai Wu Ph.D.[3],

Yumei Liu[4]†,

Jiayan Huang, Ph.D.[1]†

*Joint first authorship.

† Correspondence:

Jiayan Huang, Fudan University, 130 Dong'an Road, Shanghai, 200032, China; jiayanhuang@fudan.edu.cn

Yumei Liu, Hainan Medical University; liuyumei@hainmc.edu.cn

1 NHC Key Laboratory of Health Technology Assessment, School of Public Health, Fudan University, Shanghai 200032, China

2 Nossal Institute for Global Health, Melbourne School of Population and Global Health, University of Melbourne, Australia

3 Global Health Research Center, Duke Kunshan University, Kunshan, China

4 International School of Public Health and One Health, Hainan Medical University, Haikou, Hainan Province 571199, China

5 Harvard T.H. Chan School of Public Health, Harvard University, USA

Supplementary Material 1. Descriptions of input indicators, output indicators and environmental factors.

Category	Variable	Unit	Description
DMUs	Districts under Hainan Province	District	Haikou, Sanya, Wuzhishan, Wehchang, Qionghai, Wanning, Dingan, Tunchang, Chengmai, Lingao, Danzhou, Dongfang, Ledong, Qiongzhong, Baoting, Lingshui, Baisha, Changjiang
Input Indicators	Staff	People	Number of staff in PHCIs, include medical staff, pharmacists, nurses, technicians, and other non-medical staff
	Annual bed-days	Beds*Days	Number of available beds in PHCIs times 360 days
	Building area	Square meter	Building area of PHCIs
	Equipment above 10,000 yuan	No.	Number of equipment in PHCIs that values over 10,000 yuan
	Medical appropriation income	10,000 Yuan	Medical appropriations from basic appropriations and project appropriations
Output Indicators	Number of visits	No.	Number of patients' visits to a PHCI. This number does not include visits for NAT.
	Number of admissions	No.	Number of patients admitted to PHCIs.
	Number of discharged patients	No.	Number of patients discharged from PHCIs.
Environmental Factors	Population Density	People/sq.km	Population density of each district.
	Proportion of Aged >=65	%	Proportion of residents aged above 65 in each district
	Urbanization	%	Proportion of urban population in each district
	Proportion of Ethnic Minorities	%	Proportion of the ethnic minorities' population in each district
	Gross Domestic Product (GDP)	10,000 Yuan	GDP, the economic output, in each district
	Per capita GDP	10,000 Yuan	Economic output per person in each district

Abbreviation: DMUs, decision making units; PHCIs, primary health care institutes; NAT, nucleic acid testing.

Supplementary Material 2. Descriptive statistics for input and output variables' mean values in Hainan

Year	Input before SFA Adjustment					Output		
	Staff (People)	Annual Bed-days (Beds*360days)	Building area (Sq. meter)	Equipment above 10,000 yuan (No.)	Medical Appropriation Income (1,000 yuan)	Number of visits (No.)	Number of admissions (No.)	Number of discharged patients (No.)
2011	586	112926	36478	88	12947	607092	5852	5819
2012	654	112882	39491	106	18425	682720	5519	7630
2013	698	111390	41322	116	19728	747976	5587	5603
2014	718	114829	42580	123	21541	792531	5895	5922
2015	734	118078	43832	150	29201	796963	5914	5923
2016	768	115439	45316	176	32998	806333	5588	5706
2017	822	124900	50084	176	34430	879857	5851	5841
2018	824	119133	50775	188	33711	800907	4950	4947
2019	869	114686	51291	204	24035	829283	4737	4836
2020	921	147948	55805	247	39266	647109	2959	2930
2021	986	175943	76383	339	59288	687685	3046	2982
Mean	780	124378	48487	174	29597	752587	5081	5258

Abbreviation: SFA, stochastic frontier analysis.

Supplementary Material 3. Descriptive statistics for SFA adjusted input variables in Hainan

Year	Adjusted Input				
	Staff (People)	Annual Bed-days (Beds*360days)	Building area (Sq. meter)	Equipments above 10,000 yuan (No.)	Medical Appropriation Income (1,000 yuan)
2011	718	133320	40427	136	20381
2012	786	132808	43444	154	25889
2013	831	130770	45158	164	27275
2014	849	133298	46315	174	29043
2015	865	136489	47490	199	36535
2016	899	133858	48896	224	40369
2017	951	143334	53648	223	41620
2018	955	138035	54335	234	40712
2019	1000	133126	54798	248	31344
2020	1035	170759	61089	315	47162
2021	1107	193841	80103	380	65955
Mean	909	143604	52337	223	36935

Abbreviation: SFA, stochastic frontier analysis.

Supplementary Material 4. Descriptive statistics for environmental factors in Hainan

Year	Total Population (10000 people)	Population Density (people/sq.km)	Proportion of Aged >=65 (%)	Urbanization (UP/TP) (%)	Proportion of Ethnic Minorities (%)	GDP (10000 yuan)	Per Capita GDP (10000 yuan)
2011	48.74	256.55	8.08	50.50	16.44	1,396,506.67	28,785.77
2012	49.25	259.23	8.07	51.60	16.52	1,584,183.83	32,332.73
2013	49.74	261.78	8.10	52.74	16.70	1,762,422.61	35,609.35
2014	50.19	264.18	8.11	53.76	16.68	1,935,051.78	38,729.49
2015	50.60	264.73	8.13	55.12	16.77	2,050,835.67	40,695.86
2016	50.95	268.18	8.14	56.78	16.83	2,233,333.89	43,986.45
2017	51.43	270.70	8.14	58.04	16.86	2,471,023.50	48,273.02
2018	51.90	273.20	8.15	59.06	16.75	2,674,778.50	51,770.58
2019	52.48	274.59	9.48	59.23	16.73	2,949,405.56	56,510.22
2020	56.23	294.17	10.43	60.27	16.60	3,072,149.39	55,100.93
2021	56.68	296.24	10.78	60.97	16.44	3,594,643.72	63,673.86
Mean	51.65	271.23	8.69	56.19	16.67	2,338,575.92	45,042.57

Abbreviation: GDP, gross domestic product

Supplementary Material 5. Sensitivity analyses discarding one input indicator at one time: Efficiency Scores of PHCIs in Hainan 2011-2021 by year.

Year	Staff			Annual bed-days			Building area			Equipment above 10,000 yuan			Medical appropriation income		
	TE	PTE	SE	TE	PTE	SE	TE	PTE	SE	TE	PTE	SE	TE	PTE	SE
2011	0.7737	0.9070	0.8599	0.7786	0.9059	0.8651	0.8059	0.8943	0.9024	0.7969	0.9265	0.8666	0.8101	0.9265	0.8799
2012	0.7773	0.8845	0.8829	0.7092	0.8367	0.8580	0.7748	0.8650	0.8973	0.7465	0.8748	0.8639	0.7894	0.8933	0.8893
2013	0.7707	0.8853	0.8723	0.7152	0.8353	0.8611	0.8002	0.8924	0.8950	0.7705	0.8977	0.8631	0.7748	0.9006	0.8646
2014	0.7480	0.8678	0.8639	0.7047	0.8245	0.8546	0.7805	0.8751	0.8890	0.7403	0.8781	0.8477	0.7535	0.8833	0.8579
2015	0.7947	0.9032	0.8810	0.7666	0.8796	0.8644	0.8255	0.9198	0.8983	0.7775	0.8746	0.8886	0.8031	0.9230	0.8730
2016	0.8270	0.9238	0.8960	0.8116	0.9183	0.8762	0.8564	0.9503	0.8987	0.7977	0.9116	0.8747	0.8482	0.9512	0.8905
2017	0.7966	0.9230	0.8664	0.8080	0.9176	0.8807	0.8516	0.9573	0.8885	0.8194	0.9040	0.9005	0.8139	0.9444	0.8608
2018	0.8543	0.9611	0.8901	0.8227	0.9107	0.9014	0.8711	0.9634	0.9040	0.8413	0.9406	0.8925	0.8423	0.9659	0.8729
2019	0.8619	0.9771	0.8835	0.8314	0.9372	0.8776	0.8556	0.9799	0.8733	0.8512	0.9481	0.8935	0.8572	0.9788	0.8761
2020	0.8066	0.9468	0.8552	0.7843	0.9388	0.8360	0.8324	0.9597	0.8713	0.7939	0.9179	0.8639	0.7606	0.9340	0.8173
2021	0.7408	0.9340	0.7957	0.7452	0.9266	0.8056	0.7689	0.9248	0.8286	0.7436	0.8879	0.8336	0.7391	0.9335	0.7915
Mean	0.7956	0.9194	0.8679	0.7707	0.8938	0.8619	0.8203	0.9256	0.8860	0.7890	0.9056	0.8717	0.7993	0.9304	0.8613

Abbreviation: PHCIs, primary health care institutes; DMU, decision making unit; SFA, stochastic frontier analysis; TE, technical efficiency; PTE, pure technical efficiency; SE, scale efficiency.

Supplementary Material 6. Sensitivity analyses discarding one output indicator at one time: Efficiency Scores of PHCIs in Hainan 2011-2021 by year.

Year	Number of visits			Number of admissions			Number of discharged patients		
	TE	PTE	SE	TE	PTE	SE	TE	PTE	SE
2011	0.5109	0.7706	0.6730	0.8199	0.9273	0.8893	0.8188	0.9265	0.8890
2012	0.5040	0.7715	0.6275	0.7864	0.8933	0.8848	0.7891	0.8931	0.8893
2013	0.5063	0.7434	0.6492	0.7982	0.8997	0.8885	0.8007	0.9003	0.8906
2014	0.5421	0.7531	0.6722	0.7792	0.8832	0.8835	0.7799	0.8828	0.8847
2015	0.5637	0.7805	0.6814	0.8261	0.9238	0.8953	0.8261	0.9235	0.8956
2016	0.4914	0.7943	0.6112	0.8563	0.9539	0.8958	0.8488	0.9500	0.8923
2017	0.4709	0.8252	0.5782	0.8515	0.9573	0.8885	0.8506	0.9567	0.8881
2018	0.4294	0.8056	0.5567	0.8742	0.9659	0.9053	0.8742	0.9658	0.9054
2019	0.4540	0.8111	0.5779	0.8700	0.9854	0.8834	0.8695	0.9851	0.8832
2020	0.4969	0.8393	0.6077	0.8325	0.9595	0.8716	0.8327	0.9596	0.8718
2021	0.4354	0.8397	0.5229	0.7668	0.9409	0.8164	0.7691	0.9443	0.8158
Mean	0.4914	0.7940	0.6144	0.8237	0.9355	0.8820	0.8236	0.9352	0.8824

Abbreviation: PHCIs, primary health care institutes; DMU, decision making unit; SFA, stochastic frontier analysis; TE, technical efficiency; PTE, pure technical efficiency; SE, scale efficiency.

Supplementary Material 7. Sensitivity analyses discarding one input indicator at one time: Efficiency Scores of PHCIs in Hainan 2011-2021 by DMU.

DMU	Staff			Annual bed-days			Building area			Equipment above 10,000 yuan			Medical appropriation income		
	TE	PTE	SE	TE	PTE	SE	TE	PTE	SE	TE	PTE	SE	TE	PTE	SE
Baisha	0.6506	1.0000	0.6506	0.6360	0.9697	0.6530	0.6645	0.9861	0.6716	0.6515	0.9491	0.6841	0.6626	1.0000	0.6626
Baoting	0.7552	1.0000	0.7552	0.8233	1.0000	0.8233	0.8184	0.9851	0.8241	0.5869	0.9764	0.5970	0.7833	0.9980	0.7838
Changjiang	0.8190	0.9987	0.8199	0.6767	0.8985	0.7459	0.8153	0.9723	0.8375	0.8033	0.9851	0.8139	0.7806	0.9969	0.7831
Chengmai	0.5403	0.5793	0.9283	0.7500	0.7836	0.9567	0.7500	0.7836	0.9567	0.7272	0.7558	0.9616	0.7416	0.7788	0.9504
Danzhou	0.9974	1.0000	0.9974	0.9905	1.0000	0.9905	0.9959	1.0000	0.9959	0.9974	1.0000	0.9974	0.9974	1.0000	0.9974
Dingan	0.8673	0.9756	0.8863	0.7357	0.8218	0.8953	0.8870	0.9801	0.9039	0.8837	0.9521	0.9258	0.8226	0.9733	0.8417
Dongfang	0.8625	0.9267	0.9199	0.8598	0.9256	0.9184	0.8630	0.9250	0.9229	0.8183	0.8781	0.9168	0.7940	0.8806	0.8810
Haikou	0.9358	1.0000	0.9358	0.8141	1.0000	0.8141	0.9312	1.0000	0.9312	0.9358	1.0000	0.9358	0.9030	1.0000	0.9030
Ledong	0.8039	0.8396	0.9421	0.8095	0.8328	0.9620	0.8150	0.8347	0.9699	0.6479	0.7024	0.9198	0.8150	0.8417	0.9584
Lingao	0.9255	0.9442	0.9780	0.8771	0.9020	0.9703	0.9255	0.9442	0.9780	0.8304	0.8491	0.9759	0.9228	0.9442	0.9751
Lingshui	1.0000	1.0000	1.0000	1.0000	1.0000	1.0000	1.0000	1.0000	1.0000	1.0000	1.0000	1.0000	0.9470	0.9841	0.9610
Qionghai	0.9685	0.9836	0.9843	0.9116	0.9173	0.9924	0.9838	0.9858	0.9976	0.9838	0.9858	0.9976	0.9838	0.9858	0.9976
Qiongzhong	0.3930	0.9273	0.4245	0.3898	0.8920	0.4384	0.4113	0.8686	0.4779	0.3971	0.8289	0.4867	0.4101	0.9268	0.4437
Sanya	0.7131	0.7988	0.8977	0.4309	0.5120	0.8420	0.7126	0.7944	0.9029	0.7143	0.7996	0.8981	0.7143	0.7996	0.8982
Tunchang	0.9069	1.0000	0.9069	0.9917	1.0000	0.9917	0.9917	1.0000	0.9917	0.9917	1.0000	0.9917	0.9855	1.0000	0.9855
Wanning	0.9618	0.9756	0.9853	0.9632	0.9756	0.9868	0.9088	0.9229	0.9814	0.9306	0.9569	0.9731	0.9618	0.9735	0.9874
Wenchang	0.5858	0.6000	0.9759	0.6603	0.6807	0.9725	0.6547	0.6787	0.9697	0.6660	0.6816	0.9794	0.6495	0.6641	0.9803
Wuzhishan	0.6341	1.0000	0.6341	0.5520	0.9760	0.5606	0.6359	1.0000	0.6359	0.6359	1.0000	0.6359	0.5127	1.0000	0.5127
Mean	0.7956	0.9194	0.8679	0.7707	0.8938	0.8619	0.8203	0.9256	0.8860	0.7890	0.9056	0.8717	0.7993	0.9304	0.8613

Abbreviation: PHCIs, primary health care institutes; DMU, decision making unit; SFA, stochastic frontier analysis; TE, technical efficiency; PTE, pure technical efficiency; SE, scale efficiency.

Supplementary Material 8. Sensitivity analyses discarding one output indicator at one time: Efficiency Scores of PHCIs in Hainan 2011-2021 by DMU.

DMU	Number of visits			Number of admissions			Number of discharged patients		
	**TE**	**PTE**	**SE**	**TE**	**PTE**	**SE**	**TE**	**PTE**	**SE**
Baisha	0.5147	1.0000	0.5147	0.6591	1.0000	0.6591	0.6643	1.0000	0.6643
Baoting	0.5300	1.0000	0.5300	0.8231	1.0000	0.8231	0.8214	1.0000	0.8214
Changjiang	0.2715	0.9000	0.2961	0.8196	0.9986	0.8206	0.8158	0.9987	0.8167
Chengmai	0.2038	0.4429	0.4664	0.7497	0.7835	0.9563	0.7488	0.7826	0.9563
Danzhou	0.9579	1.0000	0.9579	0.9970	1.0000	0.9970	0.9974	1.0000	0.9974
Dingan	0.2175	0.8163	0.2663	0.8864	0.9813	0.9024	0.8870	0.9814	0.9029
Dongfang	0.7504	0.9192	0.7871	0.8643	0.9270	0.9226	0.8551	0.9191	0.9209
Haikou	0.4180	0.4420	0.9464	0.9345	1.0000	0.9345	0.9321	1.0000	0.9321
Ledong	0.6526	0.7427	0.8509	0.8118	0.8411	0.9531	0.8129	0.8397	0.9579
Lingao	0.1457	0.7350	0.2469	0.9255	0.9442	0.9780	0.9255	0.9442	0.9780
Lingshui	1.0000	1.0000	1.0000	1.0000	1.0000	1.0000	1.0000	1.0000	1.0000
Qionghai	0.3347	0.5405	0.5850	0.9838	0.9858	0.9976	0.9838	0.9858	0.9976
Qiongzhong	0.1502	0.9257	0.1653	0.4103	0.9283	0.4432	0.4112	0.9285	0.4441
Sanya	0.2983	0.5616	0.5212	0.7137	0.7991	0.8980	0.7139	0.7988	0.8986
Tunchang	0.9149	1.0000	0.9149	0.9914	1.0000	0.9914	0.9917	1.0000	0.9917
Wanning	0.9042	0.9423	0.9569	0.9557	0.9685	0.9854	0.9622	0.9743	0.9871
Wenchang	0.2378	0.3244	0.7102	0.6652	0.6811	0.9787	0.6658	0.6814	0.9794
Wuzhishan	0.3425	1.0000	0.3425	0.6359	1.0000	0.6359	0.6359	1.0000	0.6359
Mean	0.4914	0.7940	0.6144	0.8237	0.9355	0.8820	0.8236	0.9352	0.8824

Abbreviation: PHCIs, primary health care institutes; DMU, decision making unit; SFA, stochastic frontier analysis; TE, technical efficiency; PTE, pure technical efficiency; SE, scale efficiency.

Supplementary Material 9. Efficiency Scores of PHCIs in Hainan 2011-2021 by DMU

DMU	Original (Stage 1)			SFA Adjusted (Stage 3)		
	TE	PTE	SE	TE	PTE	SE
Haikou	0.9358	1.0000	0.9358	0.9858	1.0000	0.9858
Sanya	0.7143	0.7996	0.8981	0.5912	0.8061	0.7387
Wuzhishan	0.6359	1.0000	0.6359	0.2719	1.0000	0.2719
Wenchang	0.6660	0.6816	0.9794	0.6980	0.7151	0.9751
Qionghai	0.9838	0.9858	0.9976	0.9970	1.0000	0.9970
Wanning	0.9632	0.9756	0.9868	0.9493	0.9930	0.9557
Dingan	0.8870	0.9814	0.9029	0.7944	0.9814	0.8072
Tunchang	0.9917	1.0000	0.9917	0.8612	1.0000	0.8612
Chengmai	0.7500	0.7836	0.9567	0.7437	0.8597	0.8636
Lingao	0.9255	0.9442	0.9780	0.8948	0.9441	0.9454
Danzhou	0.9974	1.0000	0.9974	1.0000	1.0000	1.0000
Dongfang	0.8644	0.9270	0.9226	0.8764	0.9848	0.8850
Ledong	0.8150	0.8417	0.9584	0.8479	0.9385	0.8915
Qiongzhong	0.4114	0.9285	0.4443	0.3127	0.9633	0.3247
Baoting	0.8233	1.0000	0.8233	0.3673	0.9891	0.3702
Lingshui	1.0000	1.0000	1.0000	1.0000	1.0000	1.0000
Baisha	0.6645	1.0000	0.6645	0.5011	0.9940	0.5033
Changjiang	0.8209	0.9987	0.8218	0.7120	1.0000	0.7120
Mean	0.8250	0.9360	0.8831	0.7447	0.9538	0.7827

Abbreviation: PHCIs, primary health care institutes; DMU, decision making unit; SFA, stochastic frontier analysis; TE, technical efficiency; PTE, pure technical efficiency; SE, scale efficiency.

Supplementary Material 10. TE scores in Hainan 2011-2021 by DMU and years

DMU	2011	2012	2013	2014	2015	2016	2017	2018	2019	2020	2021
Haikou	1.0000	1.0000	0.9590	0.8974	1.0000	0.9872	1.0000	1.0000	1.0000	1.0000	1.0000
Sanya	0.7219	0.6122	0.6042	0.5439	0.5013	0.5114	0.5704	0.5987	0.7134	0.5350	0.5913
Wuzhishan	0.2496	0.2527	0.2423	0.2775	0.3517	0.3692	0.3654	0.2474	0.2334	0.2218	0.1799
Wenchang	0.5575	0.5375	0.5236	0.4977	0.6094	0.7310	0.7857	0.8265	0.8787	0.8993	0.8310
Qionghai	1.0000	1.0000	1.0000	0.9669	1.0000	1.0000	1.0000	1.0000	1.0000	1.0000	1.0000
Wanning	0.8907	0.8916	0.9815	1.0000	1.0000	1.0000	1.0000	1.0000	1.0000	0.8389	0.8391
Dingan	1.0000	0.9453	0.8064	0.8168	0.7462	0.8008	0.8450	0.7880	0.7728	0.6102	0.6072
Tunchang	0.7422	0.9293	0.6547	0.5918	0.7374	1.0000	1.0000	1.0000	1.0000	0.9965	0.8208
Chengmai	0.9452	0.7196	0.6567	0.6158	0.6918	0.8531	0.8195	0.8024	0.8364	0.6332	0.6068
Lingao	1.0000	0.8171	0.7696	0.7623	0.7202	0.8862	0.8874	1.0000	1.0000	1.0000	1.0000
Danzhou	1.0000	1.0000	1.0000	1.0000	1.0000	1.0000	1.0000	1.0000	1.0000	1.0000	1.0000
Dongfang	1.0000	1.0000	1.0000	1.0000	1.0000	1.0000	0.7456	0.7780	0.7589	0.8934	0.4643
Ledong	0.5744	0.4663	0.6988	0.6838	0.9041	1.0000	1.0000	1.0000	1.0000	1.0000	1.0000
Qiongzhong	0.3131	0.2928	0.2648	0.2781	0.3108	0.3380	0.3800	0.3877	0.2943	0.2827	0.2973
Baoting	0.4232	0.3373	0.2644	0.2258	0.3551	0.4462	0.5137	0.5600	0.4100	0.2481	0.2561
Lingshui	1.0000	1.0000	1.0000	1.0000	1.0000	1.0000	1.0000	1.0000	1.0000	1.0000	1.0000
Baisha	0.3228	0.3627	0.3493	0.2780	0.3357	0.3723	0.4157	0.4646	0.9160	0.6954	1.0000
Changjiang	0.6158	0.6344	0.7380	0.7612	0.6650	0.7822	1.0000	0.7108	0.7518	0.6892	0.4839
Mean	0.7420	0.7111	0.6952	0.6776	0.7183	0.7821	0.7960	0.7869	0.8092	0.7524	0.7210
Range (Dif.)	0.2496 – 1 (0.7504)	0.2527 – 1 (0.7473)	0.2423 – 1 (0.7577)	0.2644 – 1 (0.7742)	0.3108 – 1 (0.6892)	0.3380 – 1 (0.6620)	0.3654 – 1 (0.6346)	0.2474 – 1 (0.7526)	0.2334 – 1 (0.7666)	0.2218 – 1 (0.7782)	0.1799 – 1 (0.8201)

Abbreviation: DMU, decision making unit; TE, technical efficiency.

a. Regions in poverty included Wuzhishan, Qiongzhong, Baoting, Lingshui, and Baisha.

Supplementary Material 11. MPI in Hainan 2011-2021 by DMU

DMU	MPI (A = B*C)	TEch (B)	EFFch (C = D*E)	SEch (D)	PEch(E)
Haikou	0.8913	0.8913	1.0000	1.0000	1.0000
Sanya	0.9887	1.0181	0.9711	0.9767	0.9943
Wuzhishan	0.9877	0.9910	0.9967	0.9967	1.0000
Wenchang	1.0123	0.9809	1.0320	1.0049	1.0270
Qionghai	1.0168	1.0168	1.0000	1.0000	1.0000
Wanning	0.9031	0.9060	0.9968	0.9968	1.0000
Dingan	0.9186	0.9556	0.9612	0.9665	0.9946
Tunchang	0.9315	0.9270	1.0049	1.0049	1.0000
Chengmai	0.9237	0.9602	0.9620	0.9981	0.9638
Lingao	0.9594	0.9594	1.0000	1.0000	1.0000
Danzhou	0.8815	0.8815	1.0000	1.0000	1.0000
Dongfang	0.7844	0.8452	0.9281	0.9380	0.9895
Ledong	0.9887	0.9310	1.0619	1.0312	1.0298
Qiongzhong	0.9543	0.9609	0.9931	1.0205	0.9731
Baoting	0.9087	0.9379	0.9689	0.9782	0.9905
Lingshui	0.9337	0.9116	1.0243	1.0174	1.0067
Baisha	1.0631	0.9237	1.1509	1.1242	1.0237
Changjiang	0.9632	0.9526	1.0111	1.0151	0.9961
Mean	0.9430	0.9407	1.0025	1.0032	0.9993

Abbreviation: DMU, decision making unit; MPI, Malmquist Productivity Index; TEch, technological change; EFFch, efficiency change; SEch, scale efficiency change; PEch, pure efficiency change.
